# Nonlinear Radon Transform Using Zernike Moment for Shape Analysis

**DOI:** 10.1155/2013/208402

**Published:** 2013-04-21

**Authors:** Ziping Ma, Baosheng Kang, Ke Lv, Mingzhu Zhao

**Affiliations:** ^1^School of Information and Technology, Northwest University, Xi'an 710120, China; ^2^School of Information and Computing Sciences, North University for Nationalities, Yinchuan 750021, China; ^3^College of Computing & Communication Engineering, Graduate University of Chinese Academy of Sciences, Beijing 100049, China; ^4^College of Computer Science and Technology, Zhejiang University of Technology, Hangzhou 310023, China

## Abstract

We extend the linear Radon transform to a nonlinear space and propose a method by applying the nonlinear Radon transform to Zernike moments to extract shape descriptors. These descriptors are obtained by computing Zernike moment on the radial and angular coordinates of the pattern image's nonlinear Radon matrix. Theoretical and experimental results validate the effectiveness and the robustness of the method. The experimental results show the performance of the proposed method in the case of nonlinear space equals or outperforms that in the case of linear Radon.

## 1. Introduction

Shape analysis methods have been broadly applied to biomedical signal processing, object recognition, image retrieval, target tracking, and so forth [1]. Moments methods [2, 3] can be referred to shape descriptors because of their good characterization in describing different shapes. The most important properties of shape descriptors achieved by different moments are invariance, including translation, rotation, scaling, and stretching, stability to noise, and completeness [4].

In the past twenty years, many attentions have been paid to the completeness property of the invariant descriptor set in pattern recognition and other similar application fields. These kinds of methods can be obtained by the following processes. Firstly, Fourier transform or Radon transform is employed to map the image into other space. Secondly, the different ideas can be conceived to construct invariant descriptors based on the information in new space. Sim et al. [5] gave a new method for texture image retrieval. They converted the images in Fourier domain and calculated modified Zernike moments to extract the texture descriptors. It is tested that the descriptor has higher accuracy comparing to Gabor, Radon, and wavelet based methods and requires low computational effort. However, it is not invariant to scale. Wang et al. [6] and Xiao et al. [7] introduced the Radon transform to Fourier-Mellin transform to achieve RST (rotation, scaling, and translation) invariance and RS invariance combined blur, respectively. In virtue of Xiao's idea, Zhu et al. [8] constructed RST invariants using Radon transforms and complex moments in digital watermarking. Similarly, the Zernike moments can be connected with Radon transform. Rouze et al. [9] described a method to design an approach by calculating the Zernike moments of an image from its Radon transform using a polynomial transform in the position coordinate and a Fourier transform in the angular coordinate. However, the proposed descriptors are only invariant to rotation. Meanwhile, in order to improve the precision of image retrieval and noise robustness, Hoang and Tabbone [10] proposed a new method similar to Xiao's descriptor to obtain RST invariance based on the Radon, Fourier, and Mellin transform.

Then, Radon transform is widely applied in many methods mainly because of its better properties in projection space [11–15]. In the projective space, a rotation of the original image results in a translation in the angle variable, and a scaling of the original image leads to a scaling in the spatial variable together with an amplitude scaling [16, 17]. Based on these properties, a rotation and scaling invariant function is easy to construct and highly robust to noise.

Enlightened by the peers' research works, we extend Radon transform to nonlinear Radon transform and propose a new set of complete invariant descriptors by applying Zernike moments to the radial coordinate of the pattern's nonlinear Radon space of an image [18–22].

The remainder of this paper is organized as follows. In Section 2, we briefly review the definition of nonlinear Radon transform and Zernike moments, and propose a new method based on Zernike moment and nonlinear Radon transform. In Section 3, the comparative experiments of the proposed approach with Hu moment invariance, Chong's method is conducted in terms of image retrieval efficiency, different noise robustness.  Section 4 concludes this paper.

## 2. Nonlinear Radon Transform and Zernike Moments

### 2.1. Nonlinear Radon Transform

The nonlinear Radon transform of an image function *f*(*x*, *y*) is defined as [10]

(1) P(r,θ)=R(r,θ){f(x,y)}=∬−∞∞f(x,y)δ(rq1−T(ψ(x,y),θ))dx dy,

where *ψ*(*x*, *y*) ∈ *L* ^2^(*D*), *q*_1_ is a real instance, *θ* denotes the angle vector formed by the function *ψ*(*x*, *y*), and *T*(*ψ*(*x*, *y*), *θ*) is a rotation function by *ψ*(*x*, *y*) with an angel of *θ* and defined by

(2) $$\begin{array}{c} T\left(\psi \left(x,y\right),\theta \right)-r^{q_{1}}=0. \end{array}$$

The nonlinear Radon transform indicates curve integral of the image function *f*(*x*, *y*) along different curves. The parameter *q*_1_ can control the shape of curve. Different curves can be obtained by the values of parameter *q*_1_ and function *ψ*(*x*, *y*).

Especially when *ψ*(*x*, *y*) = *x* and *q*_1_ = 1,  *T*(*ψ*(*x*, *y*), *θ*) = *x*cos⁡*θ* + *y*sin*θ*. This reveals that the linear Radon transform is the special case of nonlinear Radon transform. The results of different curves' Radon transform are shown in Table 1.

The nonlinear Radon transform has some properties that are beneficial for pattern recognition as outlined below.

(1) Periodicity: the nonlinear Radon transform of *f*(*x*, *y*) is periodic in the variable *θ* with period 2*π* when *ψ*(*x*, *y*) is an arbitrarily parametric inference,
  (3) $$\begin{array}{c} P\left(r,\theta \right)=P\left(r,\theta \pm 2k\pi \right). \end{array}$$
(2) Resistance: if *f*_1_(*x*, *y*) and *f*_2_(*x*, *y*) are two images with little difference when *ψ*(*x*, *y*) is arbitrarily parametric inference, the corresponding nonlinear Radon transform of *f*_1_(*x*, *y*) and *f*_2_(*x*, *y*) are as followes:
  (4) |P1(r,θ)−P2(r,θ)| ≤∬D||f1(r,θ)−f2(r,θ)|δ(rq1−T(ψ(x,y),θ))|dx dy.
(3) Translation: a translation of *f*(*x*, *y*) by a vector $\overset{⃑}{u}=(x_{0},y_{0})$ results in a shift in the variable *r* of *P*(*r*, *θ*) by a distance *d* = *x*_0_cos⁡*θ* + *y*_0_sin*θ* and equals to the length of the projection of $\overset{⃑}{u}$ onto the line *x*cos⁡*θ* + *y*sin*θ* = *r*,
  (5) $$\begin{array}{c} P\left(r,\theta \right)=P\left(r-x_{0}\cos\theta -y_{0}\sin\theta ,\theta \right). \end{array}$$
(4) Rotation: a rotation of *f*(*x*, *y*) by an angle *θ*_0_ implies a shift in the variable *θ* of *P*(*r*, *θ*) by a distance *θ*_0_ when *ψ*(*x*, *y*) is arbitrarily parametric inference,
  (6) $$\begin{array}{c} P\left(r,\theta \right)\to P\left(r,\theta +\theta_{0}\right). \end{array}$$
(5) Scaling: a scaling of *f*(*x*, *y*) by a factor of *a* results in a scaling in the variable *r* and 1/*a* of amplitude of *P*(*r*, *θ*), respectively, when *ψ*(*x*, *y*) represents ellipse or hyperbola curve,
  (7) $$\begin{array}{c} f\left(ax,ay\right)\to \frac{1}{a^{2}}P\left(ar,\theta \right). \end{array}$$

### 2.2. Zernike Moment

The radial Zernike moments of order (*p*, *q*) of an image function *f*(*r*, *θ*), is defined as

(8) $$\begin{array}{c} Z_{pq}=\frac{\left(p+1\right)}{\pi }\int_{0}^{2\pi }\int_{0}^{1}R_{pq}\left(r\right)e^{-q\hat{i}\theta }f\left(r,\theta \right)rdr\;d\theta , \end{array}$$

where the radial Zernike moment of order (*p*, *q*) is defined by the following equation:

(9) $$\begin{array}{c} R_{pq}\left(r\right)=\sum_{\begin{array}{c} k=q\\ p-k=\text{even} \end{array}}^{p}B_{p|q|k}r^{k}. \end{array}$$

With

(10) Bp|q|k ={(−1)((p−k)/2)((p+k)/2)!((p−k)/2)!((q+k)/2)!((k−q)/2)!,p−k=even0,p−k=odd.

### 2.3. NRZM Descriptor Based on Nonlinear Radon Transform and Zernike Moment

**Figure 1:**
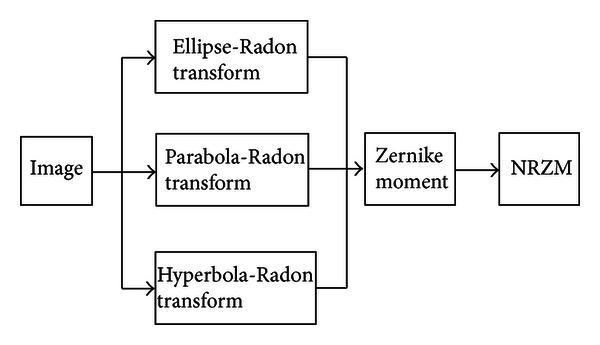
The computation process of NRZM.

The Zernike moment is carried out to be computed after the projective matrix of nonlinear Radon transform is mapped to the polar coordinate (NRZM). The computational process of our proposed method, NRZM, is illuminated in Figure 1.

Supposed $\tilde{f}(x,y)$ is the image *f*(*x*, *y*) rotated by rotational angle *β* and scaled by scaling factor *λ*, and Radon transform of $\tilde{f}(x,y)$ is given by

(11) $$\begin{array}{c} \tilde{P}\left(r,\theta \right)=\lambda P\left(\frac{r}{\lambda },\theta +\beta \right). \end{array}$$

The Zernike moments of $\tilde{P}(r,\theta )$ is

(12) Z~pq=p+1π∫02π∫01P~(r,θ)Rpq(λr)e(−i^qθ)rdr dθ=p+1π∫02π∫01λP(rλ,θ+β)Rpq(λr)e(−i^qθ)rdr dθ.

The radial Zernike polynomials *R*_*pq*_(*λr*) can be expressed as a series of *R*_*pq*_(*r*) as follows:

(13) $$\begin{array}{c} R_{pq}\left(\lambda r\right)=\sum_{k=q}^{p}R_{pk}\left(r\right)\sum_{i=q}^{k}\lambda^{i}B_{pqi}D_{pik}. \end{array}$$

The derivation process of (13) is given in the Appendix. According to (12), we have

(14) Z~pq=p+1π×∫02π∫01λP(rλ,θ+β)   ×∑k=qpRpk(r)∑i=qkλiBpqiDpike(−i^qθ)rdr dθ.

Let *τ* = *r*/*λ*,  *φ* = *θ* + *β*, (14) can be rewritten as

(15) Z~pq=p+1π×∫02π∫01λP(τ,φ)∑k=qpRpk(r)  ×∑i=qk(λiBpqiDpik)e(−i^q(φ−β))λ2τdτ dφ=p+1πei^qβ×∫02π∫01P(τ,φ)  ×∑k=qpRpk(r)∑i=qk(λi+3BpqiDpik)e−i^qφτdτ dφ=p+1πei^qβ×∑k=qp∑i=qk(λi+3BpqiDpik)    ×∫02π∫01P(τ,φ)Rpk(r)e−i^qφτdτ dφ=ei^qβ∑k=qp∑i=qk(λi+3BpqiDpik)Zpk.

Equation (15) shows that the radial Zernike moments of being rotated image can be expressed as a linear combination of the radial Zernike moments of original image. Based on this relationship, we can construct a set of rotation invariant *I*_*pq*_ which is described as follows:

(16) $$\begin{array}{c} I_{pq}=\exp\left(jqarg\left(Z_{11}\right)\right)\sum_{k=q}^{p}\left(\sum_{i=q}^{k}{Z_{00}}^{-(\left(i+3\right)/3)}B_{pqi}D_{pik}\right)Z_{pk}. \end{array}$$

Then, *I*_*pq*_ is invariant to rotation and translation.

## 3. Experimental Results and Discussions

This section is intended to test the performance of a complete family of similarity invariants introduced in the previous section for images retrieval by comparison, Chong's method presented in [12], Hu moment presented in [13]. In the experiments, the feature descriptors are calculated by

(17) $$\begin{array}{c} RZM=\left[I_{f}\left(1,0\right),I_{f}\left(1,1\right),\ldots ,I_{f}\left(M,M\right)\right]. \end{array}$$

Three subsections are included in this section. In the first subsection, we test the retrieval efficiency of proposed descriptors in shape 216 dataset. This dataset is composed of 18 shape categories with 12 samples per category, and each of every category cannot be obtained by RST transforming from any other shape from the same category. In the second subsection, we test robustness of proposed descriptors in different noisy dataset. In the third subsection, we verify the rotation invariance of the proposed method. 

### 3.1. Experiment 1

The kind of curves is changing with the controlled parameters varying. So, the retrieval efficiency is different with the controlled parameters. Many experiments are conducted to find the best parameters' values of every curve in nonlinear Radon transform, and finally the most suitable values of parameters are listed in Table 2. In the subsequent experiments, we analyze the retrieval efficiency of linear Radon transform, ellipse Radon transform, hyperbola Radon transform, and parabola Radon transform with Zernike moment, respectively, which is referred to as NZ, EPZ, HPZ, and PRZ, respectively.

**Figure 2:**
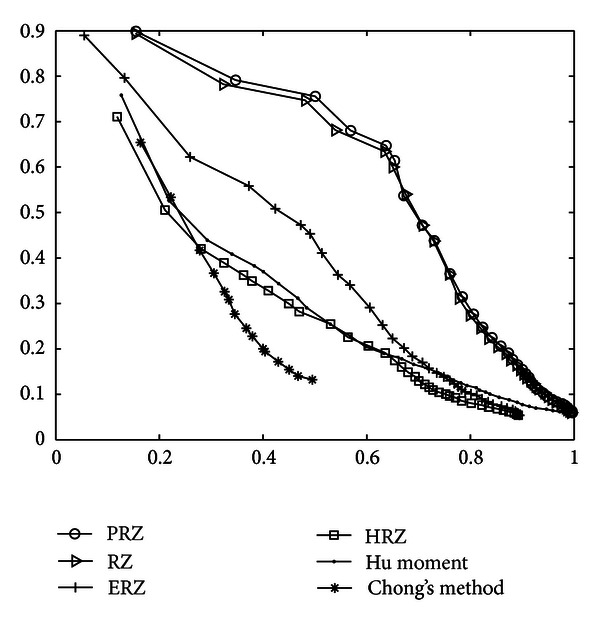
The precision-recall curve of shape 216.

In order to obtain the best retrieval efficiency of every curve Radon, the comparative precisions-recall curves in Shapes 216 are shown in Figure 2. It can be seen that the precision-recall curve of PRZ moves downward more slowly than those of others, which indicates that the retrieval efficient of PRZ is slightly higher than that of RZ while HRZ is weaker than PRZ and RZ. 

**Figure 3:**
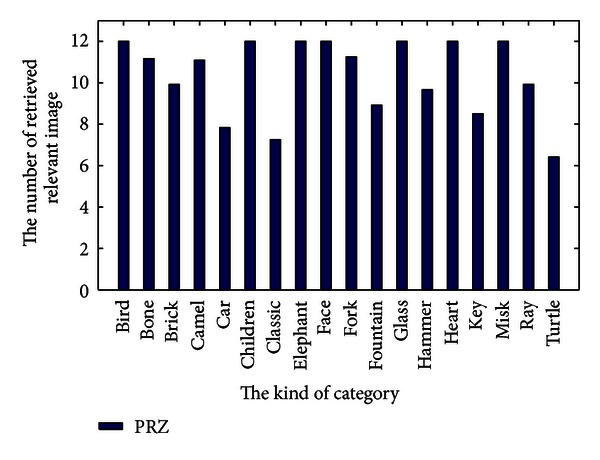
The retrieved number of every category in shape 216.

The comparative number of relevant image upon every category is a better insight into the performance of proposed method as shown in Figure 3. It is easy to see that almost the number of relevant image in every category is higher than 6, especially in bird, children, elephant, face, glass, hammer, heart, and misk.

### 3.2. Experiment 2

The robustness of the proposed descriptors is demonstrated using eight datasets added additive “salt & pepper” and “Gaussian” noise, respectively. The first seven datasets are generated from original shape 216 database, and each image is corrupted by “salt & pepper” noise with SNR varying from 16 to 4 dB with 2 dB decrements. The last one is generated from shape 216 added “Gaussian” noise with noise density = 0.01,…, 0.2. 

**Figure 4:**
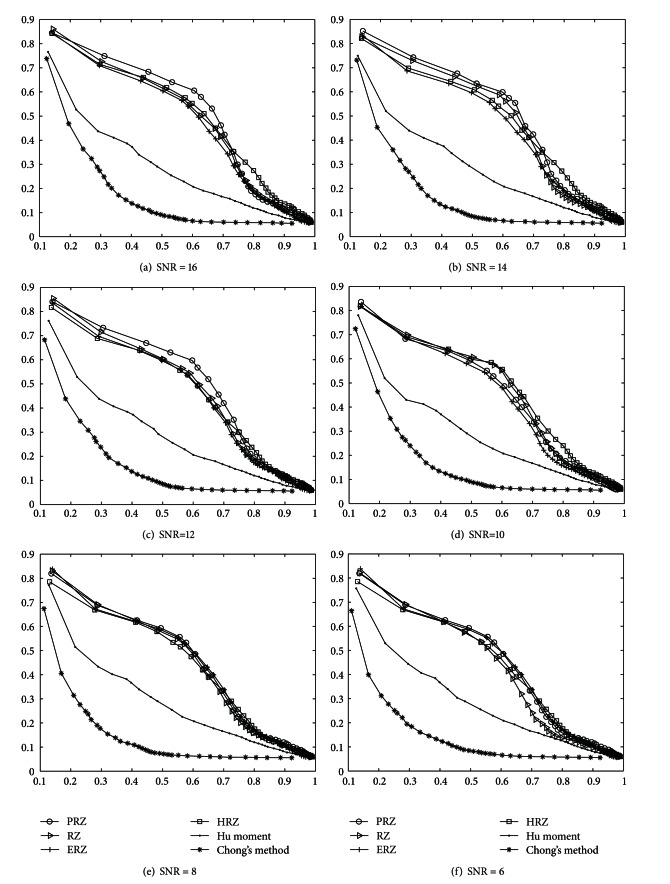

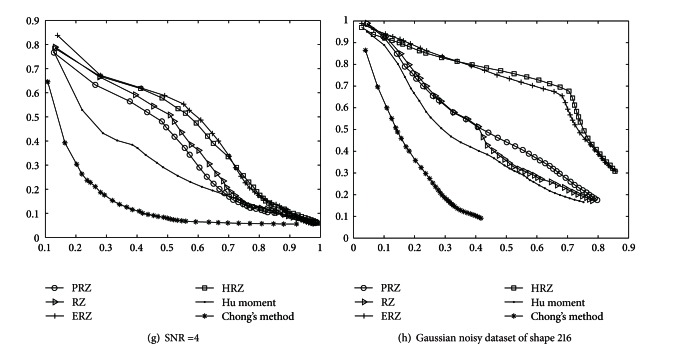
The precision upon recall curves of different descriptors on seven noisy datasets added “salt & pepper” and one “Gaussian” noisy dataset.

The retrieval experiments are conducted again in the datasets mentioned above and the precision-recall curves of comparative descriptors are depicted in Figure 4. From Figures 4(a)–4(g), it can be observed that efficiency of the PRZ and RZ are similar. It also can be seen that the PRZ and RZ descriptors have better performances than other comparative methods in “salt and pepper” noisy datasets from SNR = 16 to 8, while Hu moment and Chong's descriptors have similarly the worse performance. However, when SNR = 6 and SNR = 4, the situation has changed. The deterioration appears in the PRZ and RZ because their precision-recall curves moves downward more rapidly than those of HPZ and EPZ, while they move downward more slowly than those of Chong's method and CMI. This demonstrates that PRZ and RZ descriptor are sensitive than other nonlinear methods' descriptors when the value of SNR is low of 8 though it has the stronger robustness than Chong's method and Hu moment. In short, the impact of noise on RZ, ERZ, HRZ, and PRZ curves sometimes were little similar or sometimes differ from one to another. It is also observed that

(1) as the values of SNR decrease, the curves of all the descriptors generally move downwards;
(2) Hu moment and Chong's descriptors are very sensitive to noise, and their performance has not changed much under different levels of noise;
(3) Hu moment method has more resistance to “salt & pepper” noise than Chong's descriptors;
(4) among the RZ, ERZ, PRZ, and HRZ, the resistance of PRZ is the strongest to “salt & pepper” noise and that of RZ is close to PRZ when the values of SNR are higher than 6;
(5) PRZ is always slightly more robust to “salt & pepper” noise than RZ except for SNR = 6 and SNR = 4;
(6) EPZ and HPZ descriptors are more robust to “salt & pepper” noise than PRZ and RZ when values of SNR are higher than 6.

However, the retrieval results shown in Figure 4(h) are essentially different from those in Figures 4(a)–4(g). It is clear that ERZ and HRZ are more robust to “Gaussian” noise than other methods because their precision-recall curves are absolutely on the top of others in the “Gaussian” noisy dataset. This indicates that “Gaussian” noise can result in poor performance in the case of linear transform. In these cases, the nonlinear Radon transform should be a top priority to be employed in the proposed method. 

### 3.3. Experiment 3

**Figure 5:**
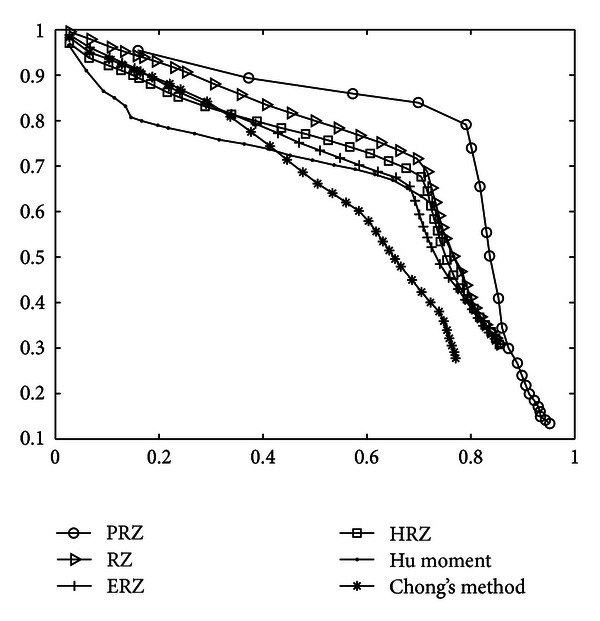
The precision-recall curves of different descriptors on rotated dataset.

The last test dataset is color objective dataset generated by choosing 7 sample images from Col and View subset. Each of the datasets is transformed by being rotated by 72 arbitrary angles (10–360) with 5 degree increment. As a result, the last dataset consists of 504 images, and the retrieval results are shown in Figure 5. From the figure, it can be concluded that the proposed descriptors are invariant to rotation, and the retrieval performance of PRZ is more efficient.

## 4. Conclusion

In this paper, we proposed a method to derive a set of rotation invariants using Radon transform and Zernike moments and extend linear Radon transform to nonlinear Radon transform. 

Comparing to linear Radon transform, the proposed method can perform better or similar. However, the numerical experiments show that different curve Radon transforms and Zernike moment perform different. In the noiseless dataset, the retrieval efficiency of PRZ is higher than comparative methods. In the “salt & pepper” noise and the PRZ consistently performs better except SNR = 6 and SNR = 4. While when SNR = 6, SNR = 4, the EPZ and HPZ are most robust than RZ. And in “Gaussian” noise dataset, the proposed method in the cases of nonlinear Radon transform is more robust to “Gaussian” noise than that in the case of linear Radon transform. Moreover, the nonlinear Radon transform can be exploited to other application fields for engineer application and recognition for the sake of the good characteristic, especially their robustness.

## Figures and Tables

**Table 1:** The diagrams of results using different curves' Radon transform.

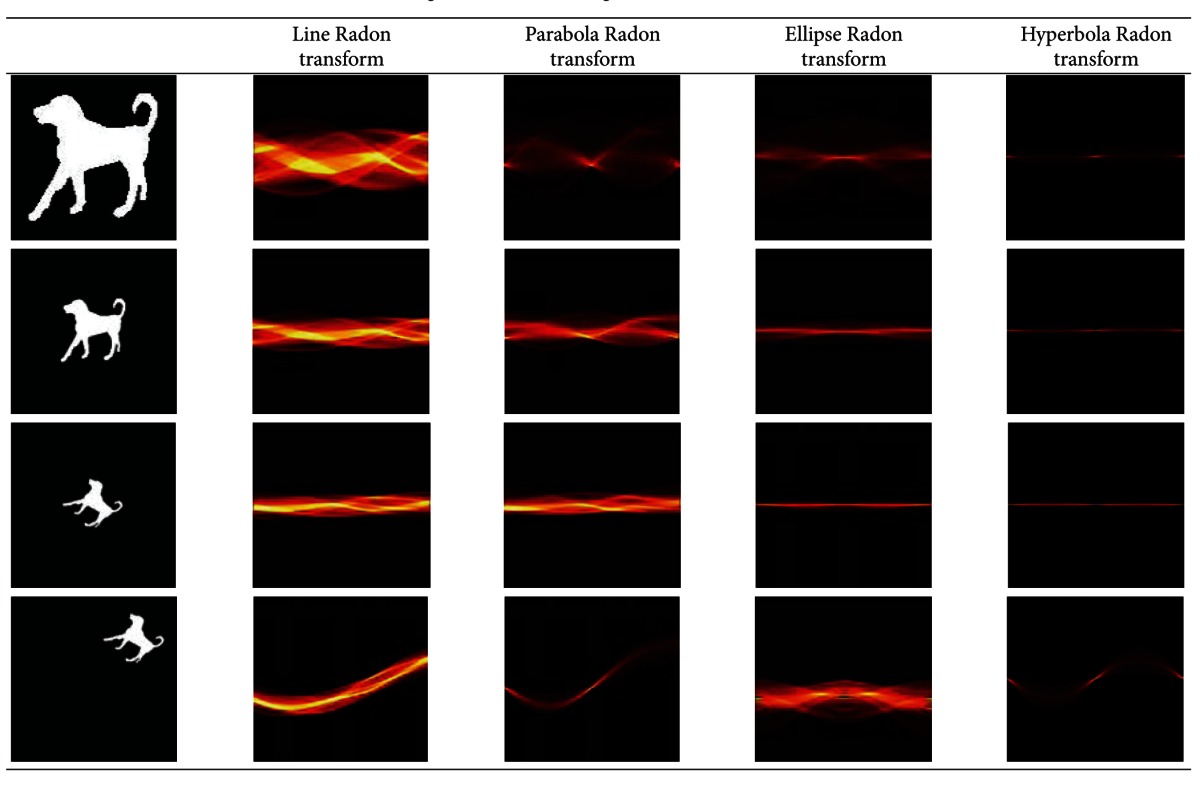

**Table 2:** The most suitable values of parameters.

The kind of curves	*q*__0__	*q*__1__
Ellipse	190/90	1
Hyperbola	350/100	2
Parabola	2000	2

## Appendix

### Proof of (13)

From (12), the radial Zernike polynomials can be expressed as a series of decreasing power of as follows:

(A.1) (Rpq(r)Rpq+1(r)⋮Rpp(r))  =(BpqqBpqq+1⋯BpqpBpq+1q+1⋯Bpq+1p⋱⋮Bppp)(rqrq+1⋮rp).

Since all the diagonal element *B*_*pii*_ are not zero, the matrix *B* is nonsingular, thus

(A.2)

## References

[1] Teng Z, He J, Degnan AJ (2012). Critical mechanical conditions around neovessels in carotid atherosclerotic plaque may promote intraplaque hemorrhage. *Atherosclerosis*.

[2] Chen SY, Zhang J, Guan Q, Liu S (2011). Detection and amendment of shape distortions based on moment invariants for active shape models. *IET Image Processing*.

[3] Wood J (1996). Invariant pattern recognition: a review. *Pattern Recognition*.

[4] Ghorbel F, Derrode S, Mezhoud R, Bannour T, Dhahbi S (2006). Image reconstruction from a complete set of similarity invariants extracted from complex moments. *Pattern Recognition Letters*.

[5] Sim DG, Kim HK, Park RH (2004). Invariant texture retrieval using modified Zernike moments. *Image and Vision Computing*.

[6] Wang X, Guo FX, Xiao B, Ma JF (2010). Rotation invariant analysis and orientation estimation method for texture classification based on Radon transform and correlation analysis. *Journal of Visual Communication and Image Representation*.

[7] Xiao B, Ma J, Cui JT (2012). Combined blur, translation, scale and rotation invariant image recognition by Radon and pseudo-Fourier-Mellin transforms. *Pattern Recognition*.

[8] Zhu HQ, Liu M, Li Y (2010). The RST invariant digital image watermarking using Radon transforms and complex moments. *Digital Signal Processing*.

[9] Rouze NC, Soon VC, Hutchins GD (2006). On the connection between the Zernike moments and Radon transform of an image. *Pattern Recognition Letters*.

[10] Hoang TV, Tabbone S (2012). Invariant pattern recognition using the RFM descriptor. *Pattern Recognition*.

[11] Deans SR (1983). *The Radon Transform and Some of Its Applications*.

[12] Hiriyannaiah HP, Ramakrishnan KR (1994). Moments estimation in Radon space. *Pattern Recognition Letters*.

[13] Galigekere RR, Holdsworth DW, Swamy MNS, Fenster A (2000). Moment patterns in the Radon space. *Optical Engineering*.

[14] Peyrin F, Goutte R Image invariant via the Radon transform.

[15] Flusser J, Suk T (1998). Degraded image analysis: an invariant approach. *IEEE Transactions on Pattern Analysis and Machine Intelligence*.

[16] Chong CW, Raveendran P, Mukundan R (2004). Translation and scale invariants of Legendre moments. *Pattern Recognition*.

[17] Zhang X, Zhang Y, Zhang J, Li X, Chen S, Chen D (2012). Unsupervised clustering for logo images using singular values region covariance matrices on Lie groups. *Optical Engineering*.

[18] Hu MK (1962). Visual pattern recognition by moments invariants. *IRE Transactions on Information Theory*.

[19] Sebastian TB, Klein PN, Kimia BB (2004). Recognition of shapes by editing their shock graphs. *IEEE Transactions on Pattern Analysis and Machine Intelligence*.

[20] http://staff.science.uva.nl/~aloi/.

[21] Zhu H, Liu M, Ji H, Li Y (2010). Combined invariants to blur and rotation using Zernike moment descriptors. *Pattern Analysis and Applications*.

[22] http://museumvictoria.com.au/bioinformatics/butter/images/bthumbliv.htm.

